# Optimisation in the Design of Environmental Sensor Networks with Robustness Consideration

**DOI:** 10.3390/s151229765

**Published:** 2015-11-27

**Authors:** Setia Budi, Paulo de Souza, Greg Timms, Vishv Malhotra, Paul Turner

**Affiliations:** 1 School of Engineering and ICT, University of Tasmania, Private Bag 87, Hobart, TAS 7001, Australia; Vishv.Malhotra@utas.edu.au (V.M.); Paul.Turner@utas.edu.au (P.T.); 2 Commonwealth Scientific and Industrial Research Organisation, 15 College Road, Sandy Bay, TAS 7005, Australia; Paulo.Desouza@csiro.au (P.S); Greg.Timms@csiro.au (G.T.)

**Keywords:** environmental sensor networks, sensor networks design, sensor networks deployment, optimisation, evolutionary algorithm, spatial regression test, gap filling, noise detection, data quality

## Abstract

This work proposes the design of Environmental Sensor Networks (ESN) through balancing robustness and redundancy. An Evolutionary Algorithm (EA) is employed to find the optimal placement of sensor nodes in the Region of Interest (RoI). Data quality issues are introduced to simulate their impact on the performance of the ESN. Spatial Regression Test (SRT) is also utilised to promote robustness in data quality of the designed ESN. The proposed method provides high network representativeness (fit for purpose) with minimum sensor redundancy (cost), and ensures robustness by enabling the network to continue to achieve its objectives when some sensors fail.

## 1. Introduction

### 1.1. Environmental Sensor Networks

Automated environmental monitoring started with simple automatic logging systems that recorded several environmental properties at predetermined intervals. These simple monitoring systems had no communication capability. They required field scientists to visit the site regularly and download the data manually. Technological advancements enabled these passive logging systems to evolve into intelligent sensor networks where each sensor node actively communicates its own observed data to nearby sensor nodes. Moreover, these interconnected sensor nodes also have a capability to process and communicate their data to a remote data centre without any operator intervention. These monitoring systems are known as Environmental Sensor Networks (ESNs) [1,2,3,4].

ESNs have a significant role to support the quality of our life on this planet. They play a part in many different areas such as agriculture, forestry, science, health and safety, insurance, mining, weather forecast, *etc*.

Production in agriculture and forestry are highly dependent on the changes in the environmental parameters (temperature, humidity, rain fall, solar radiation). Apart from the need to increase production, agricultural management should also be practiced with a degree of precision (known as Precision Agriculture) to provide an alternative and realistic means to reduce the use of potentially harmful compounds and promote sustainability. Precision Agriculture is an emerging area where ESNs play an important role [5,6,7,8].

In forestry, ESNs are also utilised for fire detection systems [9,10,11]. The networks can alarm on the origin of the fire before it is spread uncontrollably. A major forest fire can destroy thousands of hectares and incur social, environmental and economic costs.

As living beings, water and air are crucial to support our life. The quality of the water and the air which we consume and breath every day directly impact our health. The need to promote better healthcare also motivates the extensive use of ESNs to monitor the quality of both water [12,13,14] and air [15,16,17].

In the scientific field, ESNs enable us to have a better understanding of the planet on which we live. It helps us to answer many questions which could not be answered in the past and also to promote more questions which have never been asked before. The changes in climate across the earth would never be able to be identified without ESNs. Nowadays, scientists around the world have more data than before to unveil the climate change and deeply analyse its impact [18,19].

The advancements in ESNs also benefit our day to day life by providing more accurate weather information (weather report and forecast), which are crucial in certain areas like tourism and transportation. Such information is also used to support personal decisions as simple as deciding what kind of clothes to wear in the day to suit the weather.

### 1.2. ESN Design and Its Challenges

In order to have a fit for purpose ESN, design is a critical process prior to the deployment phase. There are two fundamental questions that need to be addressed: how many sensor nodes are required to fit the application purposes and where should the nodes be deployed in the Region of Interest (RoI) [20,21].

In current ESN design practice, one of the major focus for reducing costs is to minimise the total number of sensor nodes required to cover a specific RoI [22]. However, when sensors fail, the usefulness of the network degrades. The ESN no longer produces the data needed; it is not advisable, or even possible, to rely on data from such a network for decision-making. Improving robustness of ESNs is paramount.

Sensor nodes placement significantly impacts the effectiveness of an ESN and the efficiency of its operation [23]. Creating an optimised sensor node placement is not an easy problem, and it has been proven to be NP-hard (Non-deterministic Polynomial-time hard) for most formulations of sensor deployment [24]. Complexity is introduced especially when dealing with the requirement to have a fully operational ESN, which meets the application purposes, with the lowest possible number of sensor nodes. Moreover, the uncertainty in a sensor’s ability to function properly, resulting from disruptions that may be caused by terrain or harsh operational conditions in outdoor environmental monitoring, introduces further complexity.

A number of studies have been carried out in the past few years with the aim of optimising the placement of sensor nodes. As an overview, Younis and Akkaya presented a comprehensive survey of strategies and techniques in sensor networks deployment prior to 2008 [24,25]. Cheng *et al.* compared six different ESN deployment strategies (single static sink, mobile data sink, multiple data sink/clustering, non-uniform energy, non-uniform placement, non-uniform traffic) to determine the maximum achievable networks lifetime with minimum deployment cost. They mainly considered communication cost as the main contributor in power consumption; excluded some other factors such as sensing and processing cost. Linear programming is employed in their work to find the maximum lifetime for a given scenario [26]. Gribaudo *et al.* employed the modelling power of Interacting Markovian Agent to evaluate the performance of on-off strategies in a sensor network; where sensors are distributed in a continuous finite geographical area (based on a known spatial Poisson density) [27]. Bhondekar *et al.* constructed a hypothetical application involving deployment of three types of sensors, which measure three different environment properties for different applications, on a two dimensional field. A Genetic Algorithm (GA) is employed to optimise application specific parameters, connectivity parameters and energy parameters formulated into a single fitness function (weighted sum approach) [28]. Aziz *et al.* formulated the coverage problem in the deployment of ESN as an optimisation issue and Particle Swarm Optimisation (PSO) is employed to discover the near optimum placement of the sensor nodes (within a two dimensional square area) which leads to the maximum possible coverage. The fitness for each discovered solution in every iteration is evaluated based on Voronoi Diagram (VD) [29]. Beccuti *et al.* utilised Petri Nets and Markov Decision Process to solve the problem of finding a good trade off between the power consumption and the sensor network reliability [30]. Akbarzadeh *et al.* proposed a method to find a near optimum sensor deployment with the main objective to maximise the coverage within three dimensional space. Distance, orientation, and visibility are included in their work as constraint factors. The study utilised Covariance Matrix Adaptation Evolution Strategy (CMA-ES) and linked it to a Geographical Information System (GIS) to provide essential environmental data such as elevation of the RoI and obstacles in the area, to compute the fitness of individuals. A mountainous area in North Carolina was selected as the case study in their work [31]. Fan *et al.* worked on the deployment strategy that meets the coverage requirement of a sensor network by using a minimum number of sensor nodes, which are randomly and uniformly deployed in the monitored field (two spatial dimensions). Two deployment strategies are proposed in their work: Expected-area Coverage Deployment (ECD) and BOundary Assistant Deployment (BOAD) [22]. Senel *et al.* proposed an approach to increase the network connectivity and better spread the load among the relay nodes in sensor networks. The study is inspired by the behavior of a spider, which establishes a web for spanning gaps between objects [32]. Kulkarni and Venayagamoorthy presented a brief review for the application of Particle Swarm Optimisation (PSO) in sensor networks, including the networks deployment. For sensor networks deployment, the survey focused on optimising the coverage, connectivity, and energy consumption in three different kind of deployments: stationary sensor node deployment, mobile sensor node deployment, and base station deployment [33]. Another approach to deploy sensor nodes within three spatial dimensions is proposed by Unaldi *et al.* Bresenham’s line of sight (LOS) algorithm is included in the fitness function while maximising the coverage of sensor networks using a GA [34]. Mamun provided detailed descriptions of existing topologies in wireless sensor networks, including a comparative discussion of the performance of different topologies [35]. D’Este *et al.* proposed an automated method for generating and combining cost and benefit values in the deployment of sensor networks in coastal regions. The work was focused on the marine environment of Australia and used the Tasmanian Marine Analysis Network (TasMAN) project [36,37] as a case study. The sensor placement solution described in their work is called Automated Cost-Benefit Analysis (ACBA) [21]. Rodger suggested a fuzzy multi-sensor data fusion Kalman model to support the Integrated Vehicle Health Maintenance System (IVHMS) based on fault detection and feedback [38]. Mansouri *et al.* worked on the deployment of an ESN that involved three types of sensors, to measure three different environmental properties for different applications, on a two dimensional space. GA is employed to optimise application specific parameters, connectivity parameters and energy consumption parameters; using a single fitness function (weighted sum approach) [39]. Banimelhem *et al.* proposed a GA-based approach to extend the network lifetime by finding the near optimum location of nodes which would reduce the required communication energy. This method acts as a refinement of the existing clustering protocol, Low Energy Adaptive Clustering Hierarchy (LEACH), in which the sensor nodes are organised into clusters in order to reduce the amount of energy consumed in the communication between the nodes. In their work, they also assumed that the sensor nodes were mobile and deployed on a two dimensional space [40]. Ayinde and Barnawi also proposed another strategy in ESN deployment which would prolong the network lifetime while still satisfying a cost budget and a minimum required connectivity. They named their technique Enhanced Lifetime Deployment with Cost Constraints (ELDwCC), which is developed based on Differential Evolution (DE). DE is employed to navigate around the search space to deploy nodes in three spatial dimensions [41]. Cerotti *et al.* presented the implementation of Markovian Agent models in the deployment of wireless sensor network for forest fire monitoring system [42]. Rodger and George proposed an optimisation algorithm which provide set of solutions given certain probabilities and impacts of risks to sustainability [43]. Lanza-Gutierrez and Gomez-Pulido considered energy efficiency, coverage, and reliability in the deployment of sensor nodes within three dimensional space. Their objectives were to reduce the energy cost, increasing the network life time, maximising the amount and diversity of the information provided by the network. Five different optimisation techniques were employed and compared in their work: Multiobjective Evolutionary Algorithm Based on Decomposition (MOEA/D), Non-dominated Sorting Genetic Algorithm-II (NSGA-II), Strength Pareto Evolutionary Algorithm 2 (SPEA2), Multi-Objective Artificial Bee Colony (MO-ABC), and Multi-Objective Firefly (MO-FA) [44]. Rebai *et al.* focused on maximising the coverage of the sensing area and maintaining the connectivity between sensor nodes while reducing the number of sensor nodes deployed within a two dimensional space. Integer Linear Programming Model, Local Search (LS) and GA were employed in the study to find the optimal placement of sensor nodes [45].

The existing works on ESN design have focused on three objectives (or constraints): the coverage of sensing and measuring points, the network connectivity, and the energy consumption, which would have direct impact on the lifetime of the networks. From the current literature, we discovered that network representativeness and data quality have not been addressed sufficiently as crucial issue in the design of an ESN. It is true that adding more sensor nodes in the RoI will certainly boost the data quality and robustness of the ESN, yet redundancy in the placement of sensor nodes would also introduce an undesirable increase in deployment and maintenance costs. Further, the existing works only focused on the spatial dimension and none of the aforementioned approaches considered temporal data to optimise sensor node placement.

### 1.3. Our Work

We address the issues in the deployment of an ESN and considers data quality issues (to promote robustness) while designing the placement of sensor nodes in two spatial dimensions. We also introduce temporal data as a third dimension. In this work, we are focusing our experiment on temperature data measured on an hourly basis.

**Figure 1:**
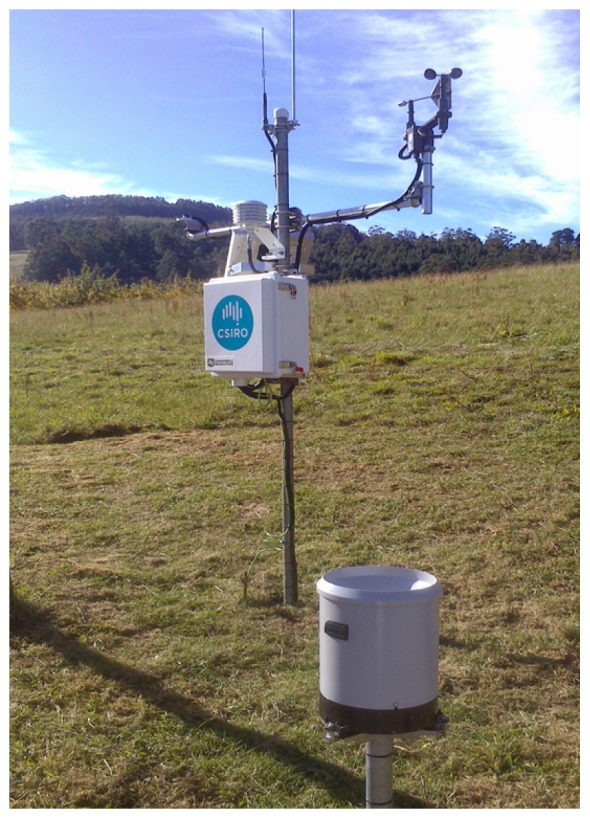
One of the weather stations operated by Commonwealth Scientific and Industrial Research Organisation (CSIRO), deployed in Geeveston (Tasmania, Australia). Similar weather stations are also deployed in other regions in Tasmania, including in South Esk. The instrument is manufactured by Campbell Scientific (model GRWS100) [46] and equipped with a set of sensors to measure several environmental properties: air temperature, relative humidity, barometric pressure, rain fall, solar radiation, wind speed and wind direction. The station also capable of sending the sensed data to a central data repository over 3G connection. Energy is provided by a solar panel.

Some fundamental assumptions underpin our work. This study is specifically dealing with the design of an ESN consisting of several weather stations acting as sensor nodes. Each node is stationary deployed and also acts as a base station, which is equipped with solar panel and telemetry (with 3G connection) to send the sensed data to a centralised data repository. Network connectivity and energy consumption are not considered as parameters to be optimised. Figure 1 shows one of our weather stations deployed in the field.

## 2. Experimental Approach

There are two main (and interrelated) components in this study: the first is optimisation of the ESN design, and the second is the data quality assessment. The optimisation mainly focused on finding the location to place each sensor node, given a certain number of sensor nodes, which will produce the best representativeness of the area. The data quality assessment focused on two common issues in ESNs: gaps and noise. Gaps in ESN data mainly occur due to sensor or communication failure, which introduce some missing values in the data. In the case of noise, the sensor still produces some data, however, the measured data does not accurately represent the actual condition. The experimental part (including simulations) in this work is written in Python programming with IPython Notebook as the platform.

### 2.1. Dataset

**Figure 2:**
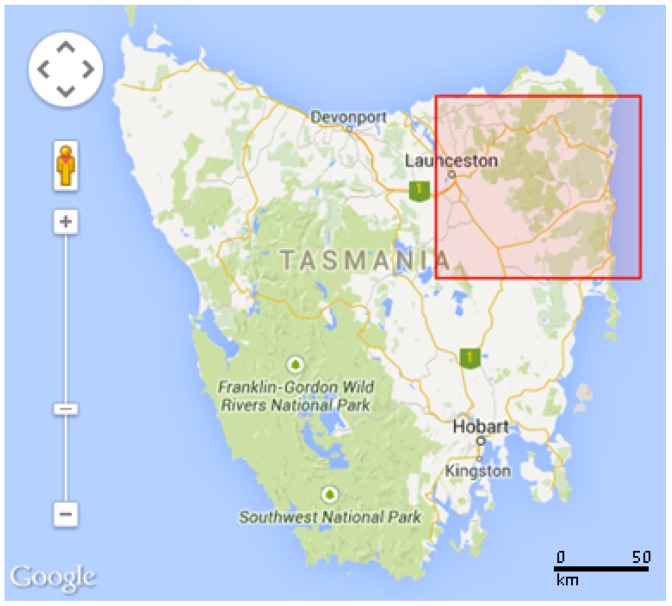
Map of Tasmania (Australia). The red colored rectangular area in the north east region indicates the Region of Interest (RoI) under study (South Esk).

The experimental part of this study was conducted using the SouthEsk Hydrological model [47] as a dataset. The model is produced by the Commonwealth Scientific and Industrial Research Organisation (CSIRO) and covers a set of environmental parameters such as temperature, relative humidity, wind speed, wind direction, and solar radiation in the North East of Tasmania (−41.0° to −42.0° latitude and 147.0° to 148.5° longitude). The region under study is presented in Figure 2.

The dataset itself is stored in Network Common Data Form (netCDF) format [48] as a multi-dimensional matrix (temporal and two spatial dimensions). The spatial area is mapped into a data grid with a size of 151 × 101. In mathematical notation, the dataset can also be expressed as a three dimensional matrix of *D*, where the first, second, and third dimensions are time, latitude, and longitude respectively. Therefore, $D_{t,i,j}$ is the environmental data measured at time index *t*, within the spatial coordinate latitude index *i* and longitude index *j*. This study will focus specifically on temperature data over 730 h, recorded on an hourly basis.

### 2.2. ESN Design Optimisation

The main focus of this component is to find the optimum placement for sensor nodes to produce the best representativeness of the RoI at minimal cost. This component covers two aspects: the representativeness measure for ESN design and the optimisation technique for placement of the sensor nodes.

#### 2.2.1. Representativeness in ESN Design

**Figure 3:**
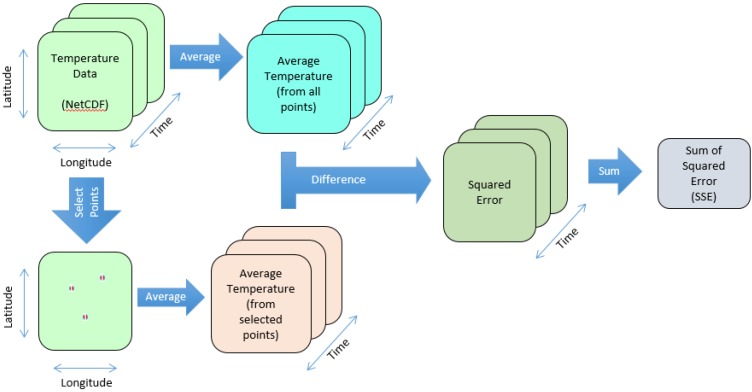
The figure shows the work flow to measure the representativeness of an ESN in respect to the Region of Interest (RoI) based on the average spatial temperature. The representativeness is calculated according to the difference between the actual average spatial temperature and the average spatial temperature measured by the deployed sensor nodes over periods of time.

In the designing process, the representativeness formulation should relate back to the purpose of the ESN deployment in the RoI. For the aim of this work, we utilised the average spatial temperature to quantify the representativeness of an ESN design as a case study. This decision is made to simplify the formulation of the representativeness. This approach follows these steps (also presented in Figure 3):

(1) The average spatial temperature is calculated from all points across the two spatial dimensions for each time step.
(2) Certain points are selected from the spatial dimensions to place the sensor nodes.
(3) The average spatial temperature is calculated from the temperature data measured by the available sensor nodes for each time step.
(4) The difference between these two average spatial temperatures is calculated for each time step and the Sum Squared Error (SSE) is produced as the result.

The average spatial temperature at a particular time index could be expressed as in Equation (1) and the design of the ESN would be defined as in Equation (2).

(1) $${\bar{D}}_{t}=\frac{\sum_{i=1}^{n_{2}}\sum_{j=1}^{n_{3}}D_{t,i,j}}{n_{2}n_{3}}$$

where: *t*is the time index*i*is the latitude index*j*is the longitude index$n_{2}$is the length of the second dimension (latitude)$n_{3}$is the length of the third dimension (longitude)$D_{t,i,j}$is the temperature data at time, latitude, and longitude index of t,i,j respectively$\bar{D_{t}}$is the average spatial temperature at time index *t*

(2) $$\begin{array}{cc} sn & =(lat,lon)\\ \vec{SN} & =(sn_{1},sn_{2},\ldots ,sn_{z}) \end{array}$$

where: snis the location of a particular sensor node which is represented as a tuple of latitude and longitude*z*is the number of sensor nodes included in the ESN design$\vec{SN}$is the ESN design which is defined as a vector of sensor nodes (sn)

The temperatures and the average spatial temperature measured by the sensor nodes in an ESN design could be expressed in Equation (3). Furthermore, the representativeness of an ESN design would be defined as the Sum Squared Error (SSE) between the average spatial temperature and the average temperature produced by the ESN over time as described in Equation (4); where a lower SSE indicates a better representativeness to the RoI.

(3) $$\begin{array}{cc} D_{t,\vec{SN}} & =D_{t,lat_{1},lon_{1}},D_{t,lat_{2},lon_{2}},\ldots ,D_{t,lat_{z},lon_{z}}\\ {\bar{D}}_{t,\vec{SN}} & =\frac{\sum D_{t,\vec{SN}}}{z} \end{array}$$

(4) $$SSE=\sum_{t=1}^{n_{1}}{\left({\bar{D}}_{t}-{\bar{D}}_{t,\vec{SN}}\right)}^{2}$$

where: $D_{t,\vec{SN}}$is a vector of temperature data measured by all sensor nodes at time index *t*${\bar{D}}_{t,\vec{SN}}$is the average spatial temperature measured by all sensor nodes at time index *t**z*is the number of sensor nodes included in the ESN design$n_{1}$is the length of the first dimension (time)SSEis the sum squared error of the average spatial temperature

#### 2.2.2. Optimisation Problem in ESN Design

The problem of finding the sensor node locations which lead to the maximum representativeness with the minimum number of sensor nodes (and hence cost) is an optimisation problem. There is an exponential growth in search space with a larger area of deployment. We assume that we have a two dimensional space of 8 x 8, and we have one sensor node to be deployed. It means within the given two dimensional space there are $2^{8}$ possible locations to deploy the sensor node. The number of possible locations will grow exponentially with the increase in the number of sensor nodes. With four sensor nodes, there are $\left[2^{8}\times \left(2^{8}-1\right)\times \left(2^{8}-2\right)\times \left(2^{8}-3\right)\right]$ possible sensor deployment schema. In this type of deployment, the search space is considerably large with each position yielding different levels of representativeness.

There are several approaches available to solve optimisation problems, namely: Dynamic Programming, Gradient Method, Evolutionary Algorithm, *etc*. Considering the large size of the search space, this study employed an Evolutionary Algorithm (EA) as an optimisation technique. The algorithm is able to handle a large search space and is also capable of avoiding local optima during the search process. Each possible solution is treated as an individual, and a set of individuals forms a population [49,50,51]. In the present work, each individual represents a certain ESN design, which consists of a set of sensor nodes including their locations.

The optimisation process is commenced with an initial randomly generated population. Next, a set of offspring are produced from the population through the crossover operation. This process is then followed by a selection process, where every individual from the previous population and their offspring are evaluated and the best solutions chosen to form a new population (the next generation). The process runs iteratively for several generations and produces several new placement designs in each generation. At the end of the iteration process (once the algorithm has converged), the best sensor placement design will be presented as the near-optimum solution. In order to avoid premature convergence, a mutation operation is applied to escape from the local optima and to diversify the population in the next generation.

In this study, the selection process uses the representativeness of an ESN design as a fitness function in order to compare two solutions (ESN designs) and determine which one is dominant over the other. Solutions which are not dominated by any other solution, will be included in the next generation population. As a final result, the population in the last generation consists of a set of solutions (ESN design) which are better (not dominated by) any other possible solution within the search space.

The algorithm in this experiment is implemented using Distributed Evolutionary Algorithms in Python (DEAP) [52], an open source evolutionary computation framework developed at the Computer Vision and Systems Laboratory of Universite Lava, Canada. We referred to De Jong’s parameters setting [53,54,55] to run EA in our experiment. The complete parameters set is presented in Table 1.

**Table 1:** Evolutionary Algorithm (EA) parameters.

Parameter	Value
Number of generation	1000
Population size	50
Crossover probability	0.6
Mutation probability	0.001
Selection operation	NSGA2 [51]
Crossover operation	one-point crossover
Mutation operation	uniform integer mutation
Seed number	0

### 2.3. ESN Data Quality Component

In order to analyse the impact of certain data quality issues on the representativeness of the ESN, artificial gaps and noise are randomly generated in a simulation.

#### 2.3.1. Gap Simulation

Artificial gaps were introduced in the simulation to analyse their impact on the representativeness of the previously discovered optimum ESN design. A gap in this simulation is formulated as a random time period where a random sensor node does not produce any data. It could also be expressed in mathematical notation as presented in Equation (5).

(5) $$\begin{array}{cc} gap & =rand(t,sn)\\ D_{gap} & =null\\ \vec{GAP} & =gap_{1},gap_{2},\ldots ,gap_{p}\\ p & =gap\_percentage\times n_{1}\times z \end{array}$$

where: rand(t,sn)is a function to select randomly a time index and a sensor node*p*is the total number of gaps$\vec{GAP}$is the vector of gaps

The gap simulation starts by generating a certain number of random time slots which are used to represent the time points where the gap occur in the data. The number of random time slots is controlled by the gap percentage parameter. In this study, the gap percentage was in the range of 10%, 20%, 30%, and 40%. Therefore, in the case of 730 measurements, 10% of gaps mean that there will be 73 points in the data which have no value. The gaps were introduced randomly to every sensor node available in the ESN design in turn, and the representativeness was determined for every sensor node. The final representativeness value of an ESN design after the inclusion of data gaps is calculated as the average representativeness of all the nodes within the ESN.

#### 2.3.2. Gap Filling

In order to overcome the loss of representativeness through gaps in data, a gap filling technique was applied. The Spatial Regression Test (SRT) [56,57] was selected as a gap filling technique due to its capability to consider both spatial and temporal aspects when predicting the missing value. Mathematically, the SRT can be expressed as in Equation (6).

(6) $$\hat{y}=\frac{\sum_{i=1}^{n}\frac{y_{i}}{S_{i}^{2}}}{\sum_{i=1}^{n}\frac{1}{S_{i}^{2}}}$$

where: $\hat{y}$is the SRT predicted value*n*is the number of neighbouring sensor nodes$y_{i}$is the linear regression predicted value from neighbour sensor node *i*$S_{i}$is the linear regression error from neighbour sensor node *i*

Gap filling was paired with gap simulation in order to present an overview of both the impact of a certain proportion of gaps in the data to the representativeness of the ESN, and how far the gap filling technique could minimise the gap impact and promote the robustness of the ESN.

#### 2.3.3. Noise Simulation

Artificial noise were also introduced in a simulation to analyse their impact on the representativeness of an ESN design. Noise in this simulation is formulated as random time points where a random sensor node produces defective data. The number of random time slots is controlled by the noise percentage parameter. In this study, the noise percentage was in the range of 10%, 20%, 30%, and 40%. The noise simulation in this study is presented in Equation (7).

(7) $$\begin{array}{cc} noise & =rand(t,sn)\\ D_{noise} & =D_{noise}+rand\left(noise\_range\right)\\ \vec{NOISE} & =noise_{1},noise_{2},\ldots ,noise_{q}\\ q & =noise\_percentage\times n_{1}\times z \end{array}$$

where: rand(t,sn)is a function to select randomly a time index and a sensor node*q*is the total number of noises$\vec{NOISE}$is the vector of noises

Unlike gap simulation which only has one parameter, in noise simulation there is a second parameter called noise range. The actual measurement drift itself was chosen randomly within a certain range which is determined by the noise range parameter. Here, the noise range is between −5 °C and +5 °C, excludes zero. Noises were introduced into every sensor node available in the ESN design in turn, and the representativeness was then calculated for every sensor node. The final representativeness value of an ESN design after the introduction of noises is calculated as the average representativeness of all the nodes within the ESN.

#### 2.3.4. Noise Detection

Unlike gaps, noises in the data are much harder to detect. In order to overcome the issue, a temperature threshold was used as an automated data quality control. The threshold was calculated based on the average and the standard deviation of the temperature data within a six hour window (three hours before and three hours after) of the data point. The threshold was generated for each data point to be assessed according to Equation (8). The constant value of two is applied in this study in order to create a 95% of confidence interval

(8) $$threshold=\bar{x}\pm C_{x}\times S_{x}$$

where: $\bar{x}$is the average temperature data within the window period$C_{x}$is a constant value.$S_{x}$is the standard deviation of the temperature data within the window period

This noise detection technique was used in conjunction with noise simulation in order to produce an overview of the impact of a certain proportion of noises to the representativeness of the ESN, as well as the effectiveness of the automated data quality technique in identifying noise. If further data quality assessment was required, we could adopt the automated marine data quality assessment technique as presented in [58,59].

## 3. Results and Discussion

### 3.1. Optimum ESN Design

**Figure 4:**
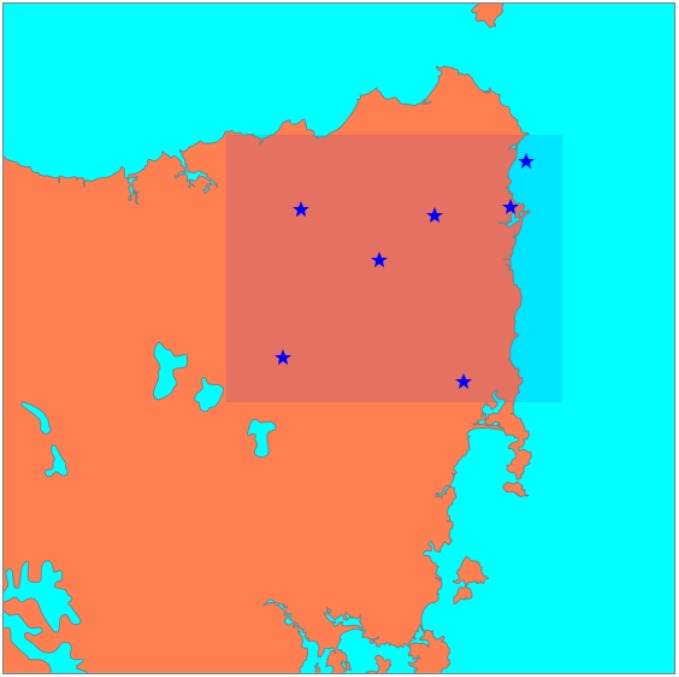
The stars indicate the optimum placement of seven sensor nodes suggested by the proposed method. The South Esk (Region of Interest in this study) is indicated by the rectangular region.

In our case study, the number of sensor nodes was set in the range from two to twenty nodes. Figure 4 presents an example result for the optimum configuration of a network consisting of seven nodes.

**Figure 5:**
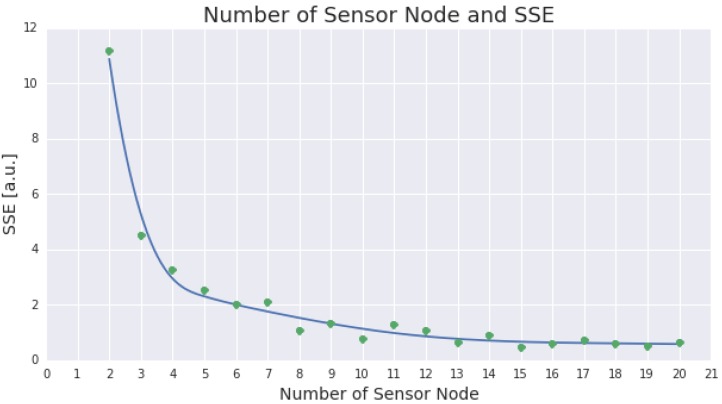
This figure presents the representativeness which could be gained from the increase in the number of deployed sensor nodes. The representativeness is measured by the difference (SSE) between the actual average spatial temperature and the average spatial temperature produced by the deployed sensor nodes over periods of time. The figure incorporates number of sensor nodes between two and twenty.

Figure 5 presents a comparison between the number of sensor nodes in each optimum ESN design and its fitness value (representativeness).

The figure indicates a significant improvement in representativeness by increasing the number of sensor nodes from two to five. Another remarkable improvement is found by adding nodes from five to ten nodes. Once the number of nodes reached thirteen, adding more nodes did not bring any significant improvement.

### 3.2. Impact of Data Gaps on ESN Design

**Figure 6:**
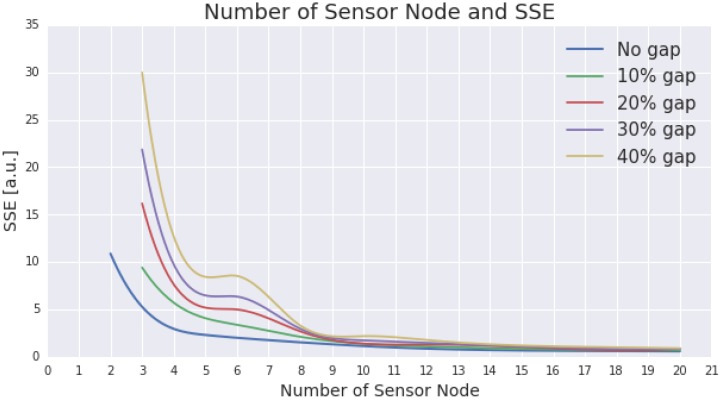
The figure presents the impact of certain degree of gaps to the performance of the proposed ESN design (sensor nodes placement). The gaps are set within the range of 10%, 20%, 30%, and 40% (illustrated by green, red, purple, and yellow lines, respectively). For comparison purposes, the performance of the ESN design without gap is also presented (using a blue line).

Figure 6 presents the result from gap simulation which incorporates 10%, 20%, 30%, and 40% of gaps. The representativeness of the optimal ESN design is also presented in the figure as a comparison of how a certain degree of gaps would impact the representativeness. The gaps within an ESN design with only two nodes will significantly degrade the representativeness. For presentation purposes, in Figure 6, gaps are introduced started with the number of sensor nodes set to three.

The figure indicates that, unsurprisingly, the more nodes that are incorporated in the design, the less the impact when gaps are introduced. Designs with less than five nodes will experience significant performance degradation once the gaps are introduced. Another notable impact also occurs in ESN with six to nine nodes. On the other hand, the gaps no longer produce any significant impact on the representativeness in an ESN design, which incorporates fifteen or more nodes.

### 3.3. Gap Filling Result

**Figure 7:**
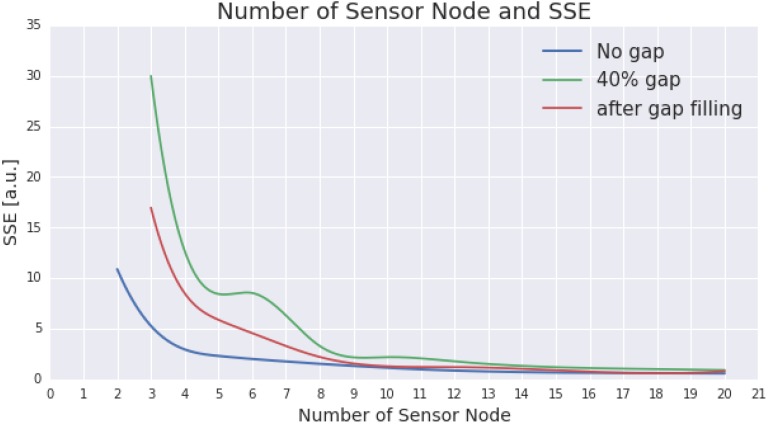
The figure presents the improvement promoted by a gap filling process (using Spatial Regression Test) to the ESN design with 40% of gaps. For comparison purposes, the performance of the ESN with no gap, with 40% of gap, and after the gap filling process are illustrated using blue, green, and red lines, respectively.

Figure 7 presents the result following gap filling using SRT with 40% of gap occurrences. A notable improvement resulting from the gap filling technique can be seen in the design with three to nine nodes. However, the improvement seems to be less significant for a design with more than thirteen nodes.

### 3.4. Impact of Noises on ESN Design

**Figure 8:**
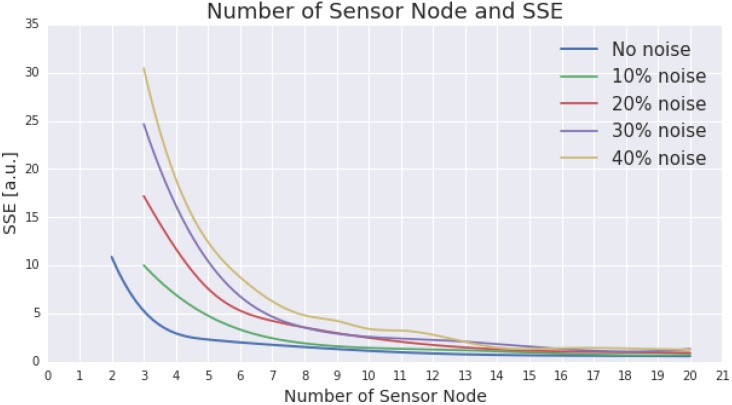
The figure presents the impact of certain degree of noise to the performance of the proposed ESN design (sensor nodes placement). The noise are set within the range of 10%, 20%, 30%, and 40% (illustrated by green, red, purple, and yellow lines, respectively). For comparison purposes, the performance of the ESN design without noise is also presented (using blue line).

Figure 8 presents the results from the noise simulation. In a similar vein to the gap simulation, the noise simulation also shows that an ESN design that incorporates more sensor nodes experiences less impact from the outlying data. Significant impact to the representativeness can be found in the designs which incorporate eight or less sensor nodes. However, after reaching fifteen nodes, having more sensor nodes in the ESN does not bring significant performance improvement when dealing with noise.

### 3.5. Noise Detection Results

**Figure 9:**
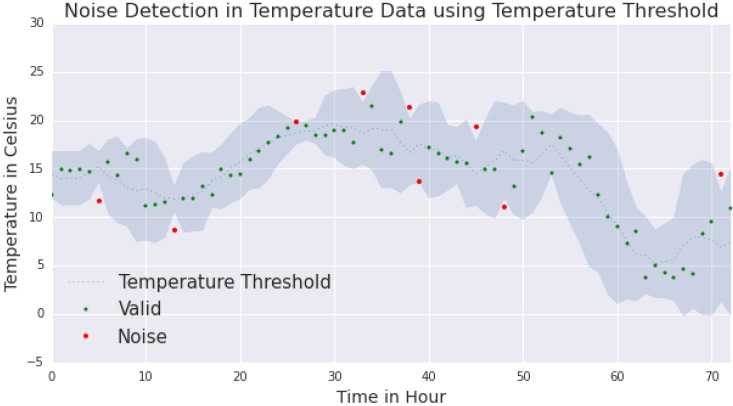
The figure indicates the detection of noise in the temperature data. Temperature Threshold (Equation 8) is applied as a noise detection technique.

Figure 9 shows the result from noise detection using the temperature threshold technique. The dots in the figure represent the measured temperature and the grey area represents the temperature threshold calculated using Equation 8. The measured data within the threshold would be considered as valid data, and are represented as green dots in the figure. On the other hand, the measured data outside the threshold would be considered as noise or invalid data, and are represented as red dots.

### 3.6. Suggestion from the Results

As a recap from the experiment, without considering any data quality issues, the ideal number of sensor nodes to be deployed in the South Esk region would be nine. However, if there is a restriction in the deployment budget, the number could be reduced down to five nodes. The deployment with 10 or more nodes would not bring any notable improvement to the representativeness of the ESN toward the RoI, and could be considered as redundant nodes deployment. On the contrary, if data quality issues (gap and noise) were to be considered in ESN design, the ideal number of nodes would be between nine and fourteen. Deploying more than fourteen nodes in the South Esk would not produce any remarkable robustness or improvement against the occurrence of data quality issues (up to 40% of gaps or noises).

## 4. Conclusions

This study proposes a design of ESNs, which is not only focused on minimising the redundancy of sensor nodes but also incorporates data quality requirements (to promote robustness). Temporal data is included during the optimisation process, in addition to two spatial dimensions. The impact of gap and noise in ESN data, including approaches to address the issues, are formalised in a simulation. The simulation presents how far the performance of an ESN, which consists of a certain number of sensor nodes, would degrade given a certain proportion of data gaps or noises. Furthermore, the simulation also demonstrates the significance of gap filling (using SRT) and noise detection (using temperature threshold) techniques in maintaining the robustness of the overall ESN performance. This information will help decision makers to determine the number and the placement of sensor nodes, which are crucial in the design of an ESN.

One challenge in our approach is that it requires an existing spatial temporal data model. Moreover, the representativeness calculations for the spatial temporal matrix are complex and the required computational time increases for larger space and longer time periods.

## Abbreviations

CSIRO: Commonwealth Scientific and Industrial Research Organisation
DEAP: Distributed Evolutionary Algorithms in Python
EA: Evolutionary Algorithm
ESN: Environmental Sensor Networks
GA: Genetic Algorithm
netCDF: Network Common Data Form
NP-hard: Non-deterministic Polynomial-time hard
NSGA2: Non Sorting Genetic Algorithm 2
RoI: Region of Interest
SRT: Spatial Regression Test
SSE: Sum Squared Error
